# Direct comparison of non-vitamin K antagonist oral anticoagulant versus warfarin for stroke prevention in non-valvular atrial fibrillation: a systematic review and meta-analysis of real-world evidences

**DOI:** 10.1186/s43044-021-00194-1

**Published:** 2021-08-11

**Authors:** Yoga Waranugraha, Ardian Rizal, Mokhamad Fahmi Rizki Syaban, Icha Farihah Deniyati Faratisha, Nabila Erina Erwan, Khadijah Cahya Yunita

**Affiliations:** 1 Department of Cardiology and Vascular Medicine, Faculty of Medicine, Universitas Brawijaya, Malang, Indonesia; 2 Faculty of Medicine, Universitas Brawijaya, Malang, Indonesia

**Keywords:** Non-vitamin K oral anticoagulant, Warfarin, Non-valvular atrial fibrillation, Meta-analysis, Real-world study

## Abstract

**Background:**

To overcome the several drawbacks of warfarin, non-vitamin K antagonist oral anticoagulants (NOACs) were developed. Even though randomized controlled trials (RCTs) provided high-quality evidence, the real-world evidence is still needed. This systematic review and meta-analysis proposed to measure the safety and efficacy profile between warfarin and NOACs in non-valvular atrial fibrillation (NVAF) patients in preventing stroke.

**Results:**

We collected articles about the real-world studies comparing warfarin and NOACs for NVAF patients recorded in electronic scientific databases such as Embase, ProQuest, PubMed, and Cochrane. The pooled hazard ratio (HR) and 95% confidence interval (CI) were estimated using the generic inverse variance method. A total of 34 real-world studies, including 2287288 NVAF patients, were involved in this study. NOACs effectively reduced the stroke risk than warfarin (HR 0.77; 95% CI 0.69 to 0.87; *p* < 0.01). Moreover, NOACs effectively lowered all-cause mortality risk (HR 0.71; 95% CI 0.63 to 0.81; *p* < 0.01). From the safety aspect, compared to warfarin, NOACs significantly reduced major bleeding risk (HR 0.68; 95% CI 0.54 to 0.86; *p* < 0.01) and intracranial bleeding risk (HR 0.54; 95% CI 0.42 to 0.70; *p* < 0.01). However, NOACs administration failed to decrease gastrointestinal bleeding risk (HR 0.78; 95% CI 0.58 to 1.06; *p* = 0.12).

**Conclusions:**

In NVAF patients, NOACs were found to be more effective than warfarin at reducing stroke risk. NOACSs also lowered the risk of all-cause mortality, cerebral hemorrhage, and severe bleeding in NVAF patients compared to warfarin.

**Supplementary Information:**

The online version contains supplementary material available at 10.1186/s43044-021-00194-1.

## Background

Atrial fibrillation (AF) puts the patients at high risk for stroke or other systemic thromboembolic events [1, 2]. Current guidelines from several cardiovascular societies recommend oral anticoagulant treatment for long-term stroke prevention strategy in AF patients [3–6]. Warfarin, a vitamin K antagonist (VKA), is an anticoagulant widely used worldwide. It effectively reduces stroke risk and mortality in AF patients [7]. However, warfarin has several drawbacks, such as the narrow therapeutic window, the requirement for stably achieved international normalized ratio (INR), the need for routine INR monitoring, the drug to food interaction, the drug to drug interaction, and drug dose adjustment [8]. A prior study revealed that an INR value below 2.0 was related to the increased risk of stroke, while an INR value above 3.0 was related to the increased bleeding risk [9]. It can be a serious problem in patients with old age, non-compliance with medication, and various comorbidities.

The non-vitamin K antagonist oral anticoagulants (NOACs), including apixaban, dabigatran, edoxaban, and rivaroxaban, were developed to overcome several drawbacks of warfarin. In the non-valvular atrial fibrillation (NVAF) population, several randomized controlled trials (RCTs) revealed that NOACs were associated with better or at least non-inferior than warfarin for systemic embolism and/or stroke prevention [10–13]. From the safety point of view, edoxaban, apixaban, and low-dose dabigatran were related to lower bleeding rates [11–13]. However, rivaroxaban and high-dose dabigatran were correlated with similar rates of bleeding [10, 11]. Even though RCTs provide good evidence, they are limited by the strict inclusion and exclusion criteria. The real-world data offer additional evidence in an extensive spectrum of the study population outside the strictly selected and controlled population involved in the RCTs [14]. Therefore, we conducted a systematic review and meta-analysis to measure the efficacy and safety profile between warfarin and NOACs in preventing stroke in NVAF patients.

## Methods

### Design

A systematic review and meta-analysis study was completed in January 2021 based on the guidance from preferred reporting items for systematic review and meta-analysis (PRISMA) [15]. We collected articles about the real-world studies comparing NOACs and warfarin in NVAF patients recorded in online databases such as Embase, ProQuest, PubMed, and Cochrane. Studies that satisfy the eligibility criteria were involved in the quality assessment of the study. The essential information was extracted only from high-quality studies. The exposure variable was anticoagulants treatment. We divided the patients into “NOACs group” and “warfarin group.” We also performed the “head to head” comparison between each NOAC (apixaban, dabigatran, edoxaban, or rivaroxaban) and warfarin. The stroke risk was our primary outcome. The secondary outcomes included the risk of: (1) all-cause mortality; (2) major bleeding; (3) intracranial bleeding; and (4) gastrointestinal bleeding. The pooled hazard ratio (HR) and 95% confidence interval (CI) were applied in determining the overall effect.

### Search strategy

Until December 2020, articles comparing the safety and efficacy of NOACs and warfarin in NVAF were collected from electronic scientific databases such as Embase, ProQuest, PubMed, and Cochrane. We used the following keywords: “non-vitamin K antagonist oral anticoagulant” or “new oral anticoagulant” or “novel oral anticoagulant’ or “NOAC,” AND “direct oral anticoagulant” or “DOAC,” AND “vitamin K antagonist” or “VKA,” AND “warfarin,” AND “dabigatran,” AND “apixaban,” AND “edoxaban,” AND “rivaroxaban,” AND “non-valvular atrial fibrillation” or “non-valvular AF” or “NVAF,” AND “stroke,” AND “cerebrovascular accident” or “CVA,” AND “death” or “all-cause death,” AND “mortality” or “all-cause mortality,” AND “major bleeding” or “major hemorrhage,” AND “intracranial bleeding” or “intracranial hemorrhage,” AND “gastrointestinal bleeding” or “gastrointestinal hemorrhage” or “GI bleeding” or "GI hemorrhage." We also collected all relevant articles through the list of references from all accessed articles or Google Scholar. We did not apply the language restriction during the initial data searching process.

### Eligibility criteria

We involved all articles which met the inclusion criteria, including: (1) cohort or real-world studies compared warfarin and NOACs in NVAF patients; (2) studies with the purpose to investigate the efficacy and/or safety profile of NOACs and warfarin in NVAF patients for stroke prevention; (3) intervention group was NOACs (apixaban, dabigatran, edoxaban, and rivaroxaban); (4) control group was warfarin; (5) availability of data about stroke, all-cause mortality, major bleeding, intracranial bleeding, or gastrointestinal bleeding; and (6) effect estimates were in HR and 95% CI. We excluded articles with one or more theses following criteria: (1) duplications; (2) not published in English; (3) involved patients with venous thromboembolism (VTE); (4) did not specify the name of the drug; (5) did not use warfarin as VKA; and (6) outcomes of interest were not reported. Two investigators reviewed all included articles. Discussion between both investigators or consultation with the third investigator was done to resolve the disagreement.

### Study quality assessment

We used the Newcastle-Ottawa scale (NOS) to evaluate the quality of the studies. It has three domains with a maximum score of 9. According to the NOS, a good quality cohort study was defined as a study with 3 to 4 stars in the selection domain, 1 to 2 stars in the comparability domain, and 2 to 3 stars in the outcome domain [16]. Two investigators performed the study quality assessment. Discrepancies between both investigators during study quality assessment were resolved by consultation or discussion with the third investigator. We only included high-quality real-world studies in this systematic review and meta-analysis.

### Data extraction

Important information about (1) name of the first author; (2) date of publication; (3) enrolment period; (4) country; (5) data source; (6) type of anticoagulants; (7) number of participants; (8) CHA2DS2-VASc score; (9) HAS-BLED score; (10) follow up period duration; (11) primary statistical model; and (12) adjusted HR and 95% CI of stroke, all-cause mortality, major bleeding, intracranial bleeding, and gastrointestinal bleeding were extracted from each study. Four investigators conducted the data extraction process.

### Statistical analysis

The meta-analysis was conducted using a combination of two software, Review Manager Version 5.3 (RevMan, Cochrane, Copenhagen, Denmark) and Comprehensive Meta-Analysis version 3.0 (CMA, New Jersey, USA). We conducted the meta-analysis based on the direction from the existing guideline [17]. We collected adjusted HR, 95% CI, and the number of participants in each group. Log HR was calculated using each study’s logarithms, while the standard error (SE) was obtained from the CI given by each study. We applied Begg’s test and Egger’s test for publication bias identification. The *p* value of < 0.05 for Begg’s test or Egger’s test represented the presence of publication bias [18–20]. The *Q* test was applied in identifying the heterogeneity among the involved studies. In the presence of heterogeneity (*p* value of heterogeneity < 0.1), we used the random-effect analysis model. On the contrary, in the absence of heterogeneity (*p* value of heterogeneity ≥ 0.1), we used the fixed-effect analysis model [21, 22]. The pooled HR and 95% CI were determined using the generic inverse variance method [23]. Statistically significant was considered if the *p* value of < 0.05. Three investigators conducted the statistical analysis process.

## Results

### Study selection and baseline characteristics

**Fig. 1:**
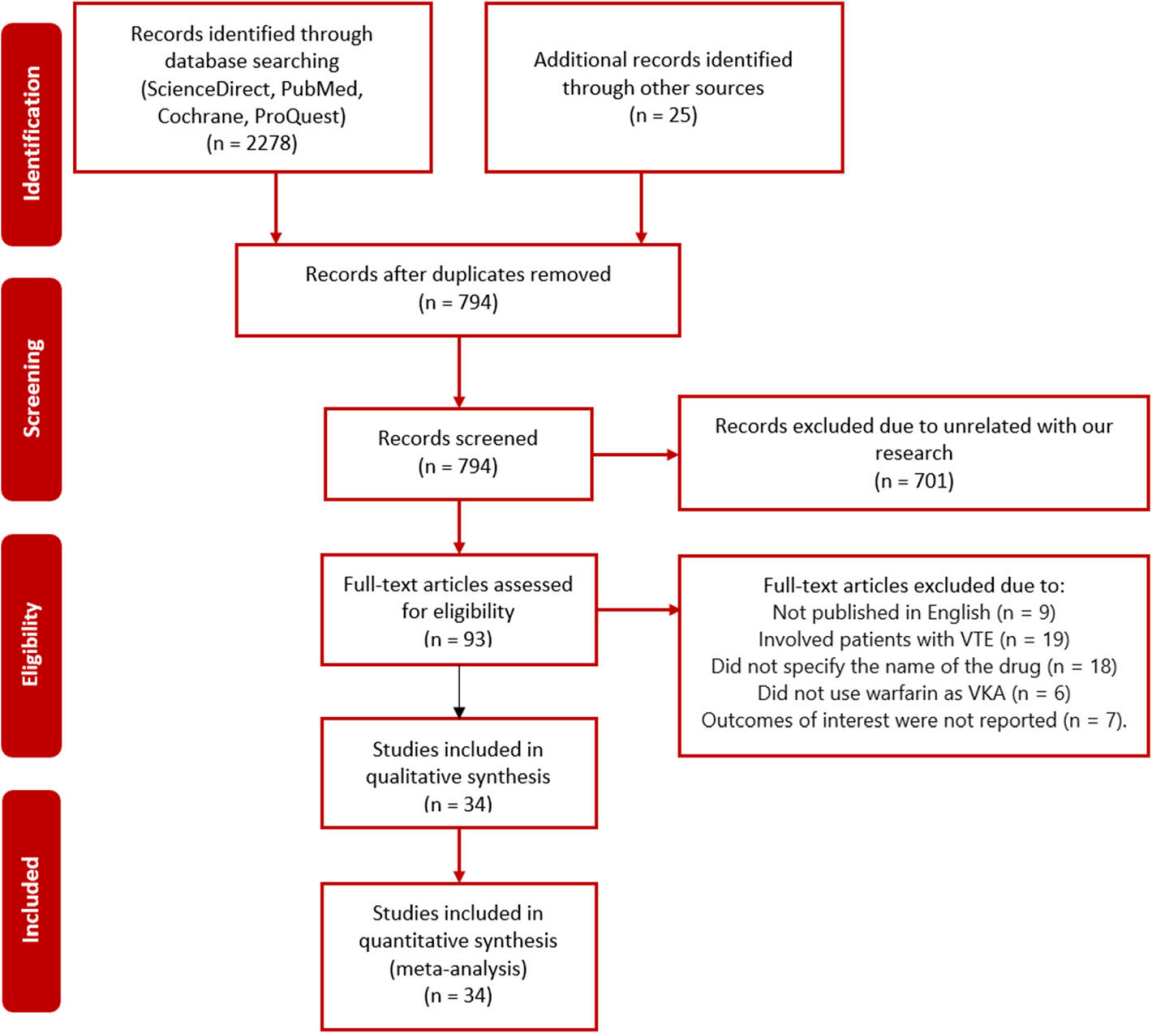
Flow diagram summarizing the selection process of included studies. *RCT* = randomized controlled trial, *VKA* = vitamin K antagonist *VTE* = venous thromboembolism

In the beginning, we had collected 2303 potentially eligible articles from electronic scientific databases. After duplicate removal, we had 794 articles. A total of 701 articles were excluded because of unrelated to our study. We performed full-text assessment in 93 studies, then a total of 59 studies were excluded due to (1) not published in English (*n* = 9); (2) involved patients with VTE (*n* = 19); (3) did not specify the name of the drug (*n* = 18); (4) did not use warfarin as VKA (*n* = 6); and (5) outcomes of interest were not reported (*n* = 7). Finally, 34 studies were involved in this study [24–57]. The study selection flowchart is presented in Fig. 1. In this study, we only involved high-quality studies assessed by NOS (Supplementary Table 1).

A total of 2287288 NVAF patients receiving apixaban, dabigatran, edoxaban, rivaroxaban, or warfarin from 34 real-world studies were involved in our meta-analysis. We involved studies that had been done in various countries in America, Asia, and Europe [24–57]. The mean CHA2DS2-VASc score ranged from 2 to 4.7 [24–30, 33, 36, 39–55, 57] while the HAS-BLED score ranged from 1.27 to 3.9 [24–26, 28–30, 33, 39, 40, 42, 46, 47, 49–55]. The primary statistical method included propensity score matching [25, 27, 31–35, 39, 41, 44, 47, 49, 50, 53, 55–57], propensity score weighting [24, 26, 28–30, 37, 38, 42, 43, 45, 46, 51, 52], and Cox proportional hazard model [36, 40, 48, 54]. The follow-up period duration was long enough [24–57]. Table 1 represents the baseline characteristics of the all included studies.

**Table 1:** Baseline characteristics of the studies

Study	Country	Enrolment period	Data source	Drugs	Participants	CHA2DS2VASc	HASBLED	Follow-up	Primary statistical method	NOS
Adeboyeje G, 2017 [24]	USA	November 2009 to January 2016	HealthCore Integrated Research Environment	A/D/R/W	44057	3.3 (mean)	2.1 (mean)	139–285 days (median)	PSW	7
Amin A, 2017 [25]	USA	January 2012 to December 2014	Center of Medicare and Medicaid Services	A/D/R/W	180020	4.4–4.7 (mean)	3.1–3.3 (mean)	196.1–203.8 days (median)	PSM	7
Bang OY, 2020 [26]	South Korea	January 2015 and November 2016	Korean Health Insurance Review and Assessment Service Database	A/D/R/W	48389	4.4–4.52 (mean)	3.5–3.54 (mean)	105–175 days (median)	PSW	8
Cha MJ, 2017 [27]	South Korea	January 2014 to December 2015	Korean National Health Insurance Service Database	A/D/R/W	34833	3.51–3.6 (mean)	NA	1.2 years (mean)	PSM	8
Chan YH, 2018 [28]	Taiwan	June 2012 to December 2016	Taiwan National Health Insurance Research Database	A/D/R/W	73074	3.26–3.89 (mean)	2.64–2.97 (mean)	0.76–1.47 years (mean)	PSW	7
Chan YH, 2019 [29]	Taiwan	June 2012 to December 2017	Taiwan National Health Insurance Research Database	A/D/E/R/W	89683	3.6 (mean)	2.6–2.7 (mean)	16 months	PSW	8
Cho MS, 2019 [30]	Korea	July 2015 to December 2016	Korean National Health Insurance Service Database	A/D/R/W	56504	3.5–3.7 (mean)	2.5–2.6 (mean)	15 months (median)	PSW	8
Coleman CI, 2017 [31]	USA	January 2012 to June 2015	Truven MarketScan	A/D/R/W	9684	5 (median)	3–4 (median)	0.5–0.6 years (mean)	PSM	8
Costa OS, 2020 [32]	USA	November 2010 to 30 September 2018	Optum Research Database	R/W	71226	3 (median)	2 (median)	2 years (median)	PSM	8
Deitelzweig S, 2017 [33]	USA	January 2013 to September 2015	Humana Research Database	A/D/R/W	32488	4.3–4.6 (mean)	2.9–3.1 (mean)	6.4–7.1 months (mean)	PSM	7
Graham DJ, 2015 [34]	USA	October 2010 to December 2012	Medicare	D/W	134414	NA	NA	180 days	PSM	8
Graham DJ, 2019 [35]	USA	October 2010 to September 2015	Medicare	A/D/R/W	448586	NA	NA	300 days	PSM	8
Halvorsen S, 2017 [36]	Norway	January 2013 to June 2015	Norwegian Patient Registry Norwegian Prescription Database	A/D/R/W	32675	2.46–3.09 (mean)	NA	143–212 days (median)	Cox proportional hazard model	7
Hernandez I, 2015 [37]	USA	October 2010 to October 2011	Medicare	D/W	9404	NA	NA	177 days (mean)	PSW	8
Hsu CC, 2018 [38]	Taiwan	January 1999 to December 2015	Taiwan National Health Insurance Research Database	D/R/W	1211	NA	NA	1.7 years (median)	PSW	7
Huybrechts KF, 2020 [39]	USA	October 2010 to September 2015	IBM MarketScan Medicare Optum Research Database	A/D/R/W	169112	3.01–3.05 (mean)	2.25–2.26 (mean)	1 year	PSM	8
Kjerpeseth LJ, 2019 [40]	Norway	July 2013 to December 2015	Norwegian Prescription Database Norwegian Patient Registry Norwegian Cause of Death Registry National Registry	A/D/R/W	30820	2.9–3.5 (mean)	2.2–2.6 (mean)	365 days	Cox proportional hazard model	7
Kohsaka S, 2020 [41]	Japan	March 2011 to July 2018	Japanese Administrative Claims	A/D/E/R/W	73989	3.8 (mean)	NA	2 years	PSM	8
Larsen TB, 2016 [42]	Denmark	August 2011 to October 2015	Danish National Prescription Registry Danish National Patient Register Danish Civil Registration System	A/D/R/W	61678	2.7 (mean)	2.2 (mean)	1.9 years (mean)	PSW	8
Lauffenburger JC, 2015 [43]	USA	October 2010 to December 2012	Truven Health MarketScan Medicare	D/W	64935	2.3–2.9 (mean)	NA	358 days (mean)	PSW	8
Lee SR, 2018 [44]	South Korea	January 2014 to December 2016	National Health Insurance Service Database	E/W	16244	3.22–3.25 (mean)	NA	0.3 to 0.9 years (median)	PSM	9
Lee SR, 2019 (1) [45]	South Korea	January 2014 to December 2016	National Health Insurance Service Database	A/D/E/R/W	24974	3 (mean)	NA	1.2 years (median)	PSW	9
Lee SR, 2019 (2) [46]	South Korea	January 2015 to December 2017	National Health Insurance Service Database	A/D/E/R/W	116804	3.54–3.6 (mean)	2.69–2.71 (mean)	1 year	PSW	9
Li X, 2017 [47]	USA	January 2012 to September 2015	Truven MarketScan IMS PharMetrics Plus Database Optum Clinformatics Data Mart Humana Research Database	A/W	76940	3.2 (mean)	2.6 (mean)	179.2–199.9 days (mean)	PSM	8
Lip YH, 2016 (1) [48]	USA	January 2013 to December 2013	Truven MarketScan Medicare	A/D/R/W	29338	2.58–3.22 (mean)	NA	90.37–127.55 days (median)	Cox proportional hazard model	7
Lip YH, 2016 (2) [49]	USA	January 2012 to December 2014	Truven MarketScan Medicare	A/D/R/W	45361	2.6–3 (mean)	2–2.2 (mean)	148.1–178.1 days (median)	PSM	7
Maura G, 2015 [50]	France	July 2011 to November 2012	French National Health Insurance Information System French Hospital Discharge Database	D/R/W	32807	2.4–3.6 (mean)	2–2.4 (mean)	80–87 days (median)	PSM	9
Mitsuntisuk P, 2020 [51]	Thailand	January 2012 to April 2018	9 Hospitals in Thailand	A/D/R/W	2055	3.25–3.86 (mean)	1.27–1.65 (mean)	1.9–2.82 years (mean)	PSW	8
Nielsen PB, 2017 [52]	Denmark	August 2011 to February 2016	Danish National Prescription Registry Danish Civil Registration System Danish National Patient Register	A/D/R/W	55644	3.3 (mean)	2.4 (mean)	2.5 years	PSW	8
Rutherford OCW, 2020 [53]	Norway	January 2013 to December 2017	The Norwegian Patient Registry The Norwegian Prescription Database	A/D/R/W	65563	2.93–3.23 (mean)	2.25–2.43 (mean)	12 months	PSM	8
Staerk L, 2017 [54]	Denmark	August 2011 to December 2015	Danish National Prescription Registry Danish Civil Registration System Danish National Patient Register	A/D/R/W	43299	2–2.2 (mean)	2.7–3.11 (mean)	204–386 days (median)	Cox proportional hazard model	7
Villines TC, 2015 [55]	USA	October 2009 to July 2013	Department of Defense Database	D/W	25586	3.4 (mean)	3.9 (mean)	217.2–297.3 days (mean)	PSM	7
Yao X, 2016 [56]	UA	October 2010 to June 2015	OptumLabs Data Warehouse	A/D/R/W	76354	3–4 (median)	2 (median)	6 months	PSM	7
Yu HT, 2018 [57]	Korea	January 2016 to December 2016	National Health Insurance Service	E/W	9537	4.2 (mean)	NA	5 months (median)	PSM	8

*A* = apixaban, *CHA2DS2-VASc* = congestive heart failure, hypertension, age 75 years or older, diabetes mellitus, previous stroke/transient ischemic attack, vascular disease, age 65 to 74 years, *D* = dabigatran, *E* = edoxaban, *HASBLED* = Hypertension, Abnormal renal or liver function, Stroke, Bleeding history, Labile international normalized ratio (INR), age ≥ 65 years, and antiplatelet Drug or alcohol use, *NA* = not available, *NOS* = Newcastle-Ottawa Scale, *PSM* = propensity score matching, *PSW* = propensity score weighting, *R* = rivaroxaban, *W* = warfarin

### Heterogeneity and publication bias

Heterogeneity was represented by a *p* value of heterogeneity of < 0.1. It was found in almost all analyses, except for the risk of: (1) stroke between edoxaban and warfarin; (2) all-cause mortality between NOACs and warfarin; and (3) intracranial bleeding between rivaroxaban and warfarin. Therefore, in almost all analyses, the random-effect analysis model was used. The *p* value of Begg’s test and Egger’s test for all analyses were > 0.05, so, no publication bias was found in this study. The assessment of heterogeneity and publication is summarized in Table 2.

**Table 2:** Summary of the outcomes of interest

Outcomes	NOACs (*n*)	Warfarin (*n*)	Model	HR	95% CI		*p* value of heterogeneity	*p* value of Begg’s test	*p* value of Egger’s test	*p*
					Lower limit	Upper limit				
Stroke										
Apixaban	256909	474732	Random	0.73	0.64	0.84	< 0.01	0.77	0.77	< 0.01
Dabigatran	345545	365144	Random	0.87	0.81	0.94	< 0.01	0.70	0.78	< 0.01
Edoxaban	46035	78185	Fixed	0.67	0.60	0.76	0.84	1.00	0.46	< 0.01
Rivaroxaban	336406	486587	Random	0.81	0.73	0.90	< 0.01	0.19	0.41	< 0.01
All NOACs	984895	1604648	Random	0.77	0.69	0.87	< 0.01	0.73	0.85	< 0.01
All-cause mortality										
Apixaban	95097	309813	Random	0.69	0.49	0.98	< 0.01	1.00	0.60	0.04
Dabigatran	216235	390118	Random	0.67	0.57	0.80	< 0.01	0.35	0.06	< 0.01
Edoxaban	16210	16785	Random	0.52	0.31	0.85	0.02	1.00	0.53	0.01
Rivaroxaban	128600	310114	Random	0.91	0.70	1.18	< 0.01	0.76	0.89	0.47
All NOACs	456142	1026830	Fixed	0.71	0.63	0.81	0.14	0.31	0.08	< 0.01
Major bleeding										
Apixaban	234818	314596	Random	0.57	0.53	0.63	< 0.01	0.42	0.20	< 0.01
Dabigatran	292539	382810	Random	0.75	0.67	0.83	< 0.01	0.27	0.15	< 0.01
Edoxaban	48875	81025	Random	0.55	0.45	0.66	0.09	0.71	0.27	< 0.01
Rivaroxaban	337030	377026	Random	0.90	0.82	0.98	< 0.01	0.38	0.06	0.01
All NOACs	913262	1155457	Random	0.68	0.54	0.86	< 0.01	0.73	0.63	< 0.01
Intracranial bleeding										
Apixaban	251901	442439	Random	0.57	0.48	0.68	< 0.01	0.71	0.06	< 0.01
Dabigatran	323015	488790	Random	0.44	0.38	0.52	< 0.01	1.00	0.14	< 0.01
Edoxaban	48875	81025	Random	0.44	0.26	0.76	< 0.01	1.00	0.06	< 0.01
Rivaroxaban	326289	439920	Fixed	0.69	0.64	0.74	0.14	0.08	0.07	< 0.01
All NOACs	950080	1452174	Random	0.54	0.42	0.70	< 0.01	0.73	0.26	< 0.01
Gastrointestinal bleeding										
Apixaban	242813	401123	Random	0.58	0.51	0.67	< 0.01	0.43	0.07	< 0.01
Dabigatran	326750	490358	Random	0.99	0.87	1.12	< 0.01	0.08	0.06	0.88
Edoxaban	48875	81025	Random	0.62	0.44	0.87	< 0.01	1.00	0.09	< 0.01
Rivaroxaban	312311	396000	Random	1.00	0.86	1.17	< 0.01	0.58	0.06	0.97
All NOACs	930749	1368506	Random	0.78	0.58	1.06	< 0.01	0.73	0.75	0.12

*CI* = confidence interval, *HR* = hazard ratio, *NOACs* = non-vitamin K antagonist oral anticoagulants

### Primary outcome

#### Stroke

**Fig. 2:**
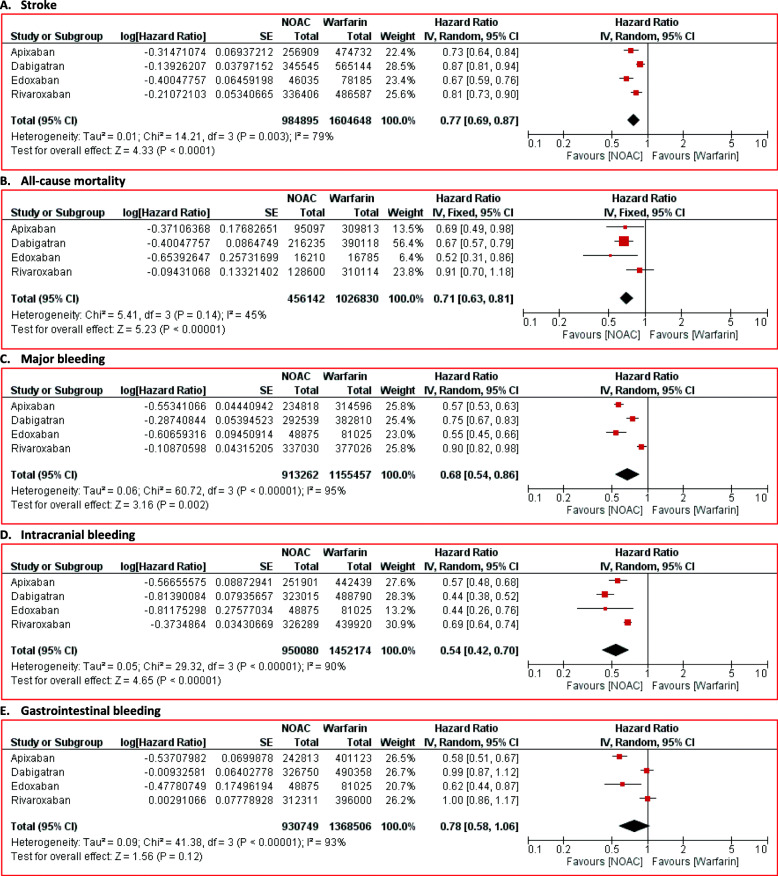
Comparison of NOACs versus warfarin for **A** stroke, **B** all-cause mortality, **C** major bleeding, **D** intracranial bleeding, and **E** gastrointestinal bleeding. CI = confidence interval; NOACs = non-vitamin K antagonist oral anticoagulants

**Fig. 3:**
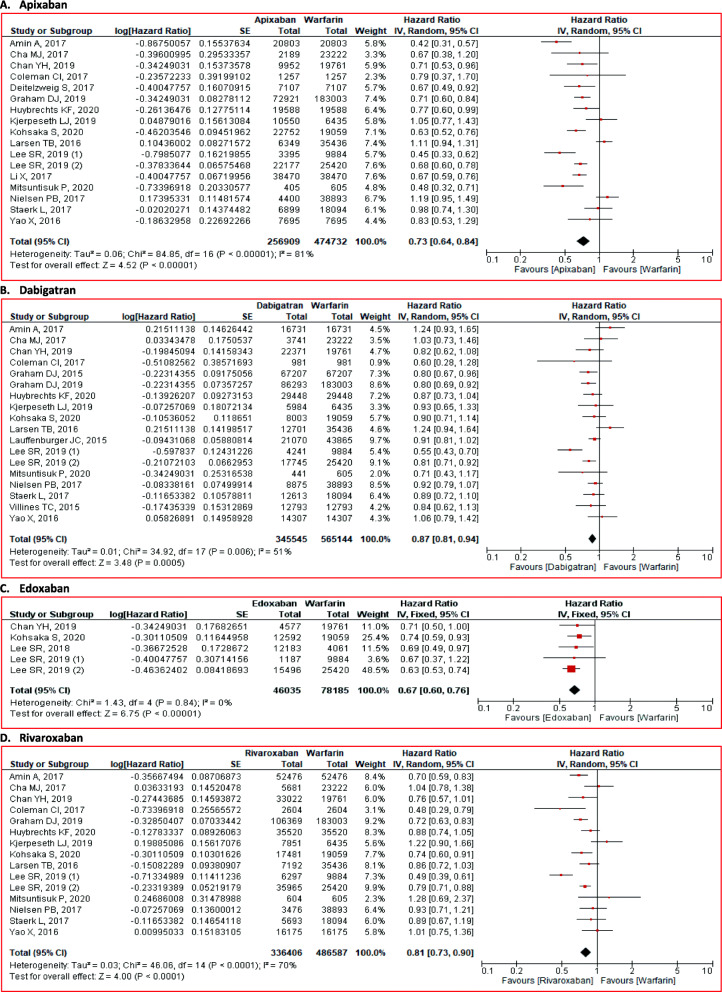
Comparison of stroke between NOACs and warfarin stratified by each agent. **A** Apixaban, **B** dabigatran, **C** edoxaban, and **D** rivaroxaban. CI = confidence interval, NOACs = non-vitamin K antagonist oral anticoagulants

Our primary outcome was the stroke risk reduction. Our result revealed that NOACs significantly reduced stroke risk in NVAF patients (HR 0.77; 95% CI 0.69 to 0.87; *p* < 0.01) compared to warfarin (Fig. 2). The subgroup analysis for the specific agent also revealed the consistent results. Apixaban (HR 0.73; 95% CI 0.64 to 0.84; *p* < 0.01), dabigatran (HR 0.87; 95% CI 0.81 to 0.94; *p* < 0.01), edoxaban (HR 0.67; 95% CI 0.60 to 0.76; *p* < 0.01), and rivaroxaban (HR 0.81; 95% CI 0.73 to 0.90; *p* < 0.01) significantly reduced stroke risk (Fig. 3).

### Secondary outcomes

#### All-cause mortality

**Fig. 4:**
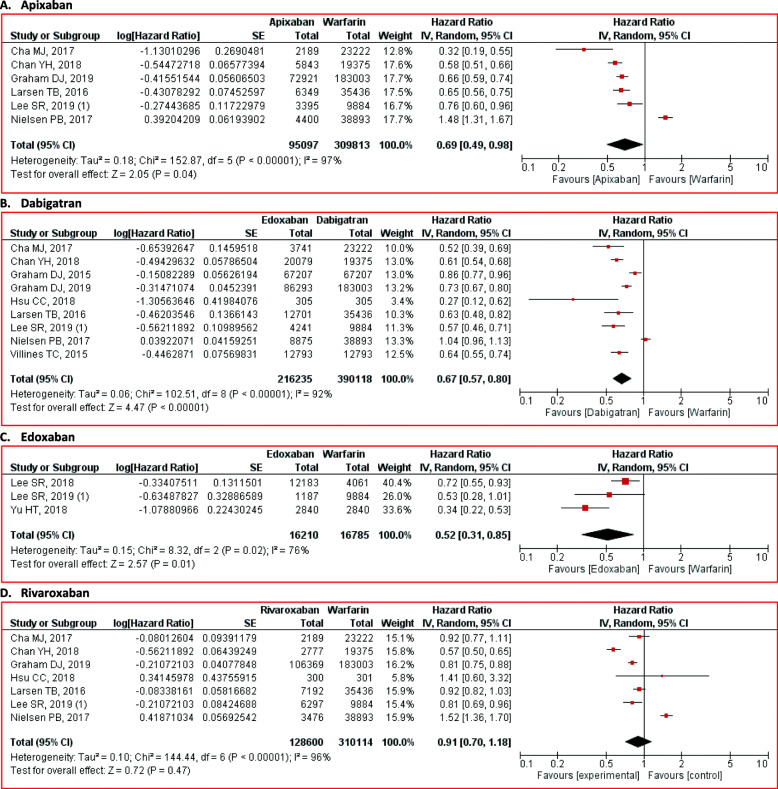
Comparison of all-cause mortality between NOACs and warfarin stratified by each agent. **A** apixaban, **B** dabigatran, **C** edoxaban, and **D** rivaroxaban. CI = confidence interval, NOACs = non-vitamin K antagonist oral anticoagulants

NOACs administration successfully reduced all-cause mortality risk than warfarin (HR 0.71; 95% CI 0.63 to 0.81; *p* < 0.01) (Fig. 2). From the subgroup analysis, we found that apixaban (HR 0.69; 95% CI 0.49 to 0.98; *p* = 0.04), dabigatran (HR 0.67; 95% CI 0.57 to 0.80; *p* < 0.01), and edoxaban (HR 0.52; 95% CI 0.31 to 0.85; *p* = 0.01) were also related to lower all-cause mortality risk than warfarin (Fig. 4). However, the all-cause mortality risk between rivaroxaban and warfarin was not different significantly (HR 0.91; 95% CI 0.70 to 1.18; *p* = 0.47) (Fig. 4).

#### Major bleeding

**Fig. 5:**
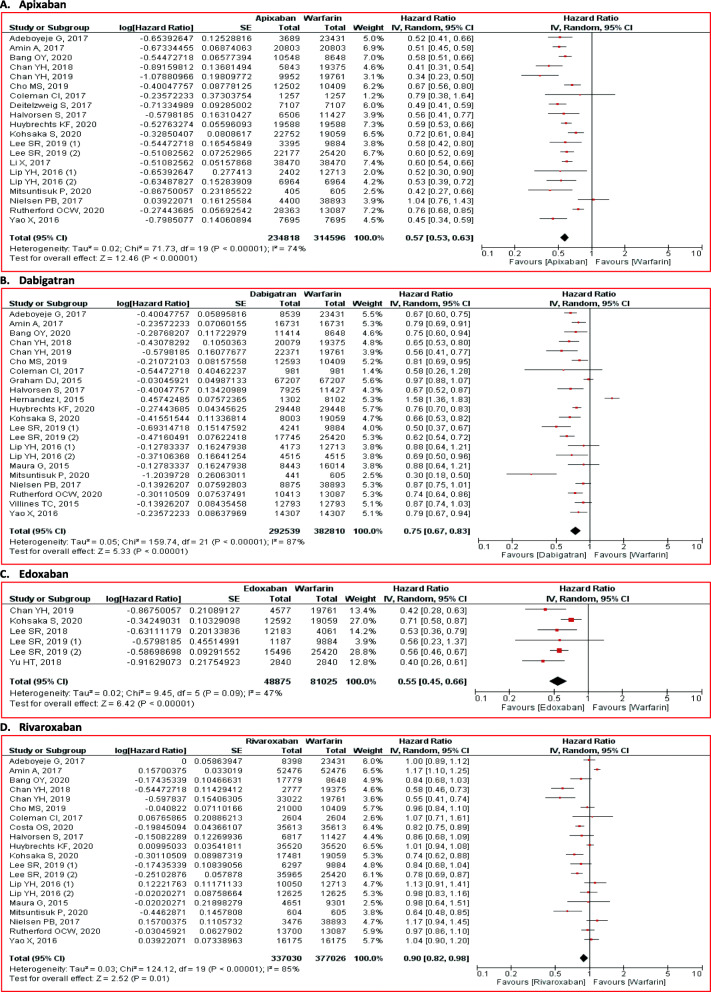
Comparison of major bleeding between NOACs and warfarin stratified by each agent. **A** apixaban, **B** dabigatran, **C** edoxaban, and **D** rivaroxaban. CI = confidence interval, NOACs = non-vitamin K antagonist oral anticoagulants

NOACs effectively reduced major bleeding risk (HR 0.68; 95% CI 0.54 to 0.86; *p* < 0.01) than warfarin (Fig. 2). The subgroup analysis also revealed the consistent results. Apixaban (HR 0.57; 95% CI 0.53 to 0.63; *p* < 0.01), dabigatran (HR 0.75; 95% CI 0.67 to 0.83; *p* < 0.01), edoxaban (HR 0.55; 95% CI 0.45 to 0.66; *p* < 0.01), and rivaroxaban (HR 0.90; 95% CI 0.82 to 0.98; *p* = 0.01) was associated with major bleeding risk reduction (Fig. 5).

#### Intracranial bleeding

**Fig. 6:**
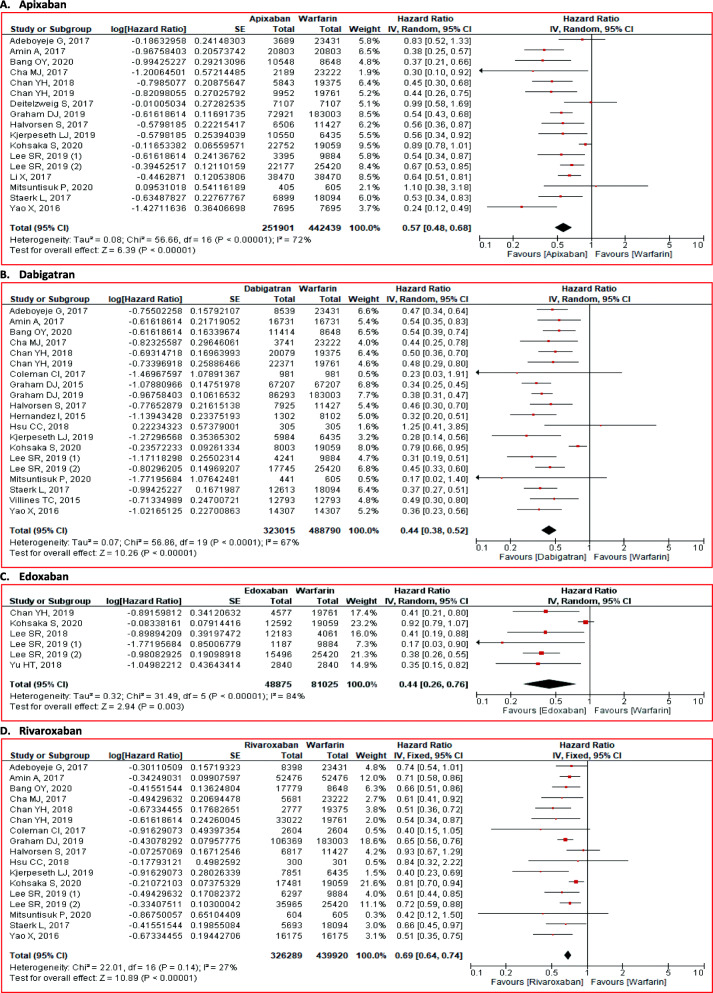
Comparison of intracranial bleeding between NOACs and warfarin stratified by each agent. **A** apixaban, **B** dabigatran, **C** edoxaban, and **D** rivaroxaban. CI = confidence interval, NOACs = non-vitamin K antagonist oral anticoagulants

NOACs administration was correlated with the lower risk for intracranial bleeding (HR 0.54; 95% CI 0.42 to 0.70; *p* < 0.01) than warfarin (Fig. 2). The similar results were also found in the agent-specific level. Apixaban (HR 0.57; 95% CI 0.48 to 0.68; *p* < 0.01), dabigatran (HR 0.44; 95% CI 0.38 to 0.52; *p* < 0.01), edoxaban (HR 0.44; 95% CI 0.26 to 0.76; *p* < 0.01), and rivaroxaban (HR 0.69; 95% CI 0.64 to 0.74; *p* < 0.01) effectively reduced major bleeding risk (Fig. 6).

#### Gastrointestinal bleeding

**Fig. 7:**
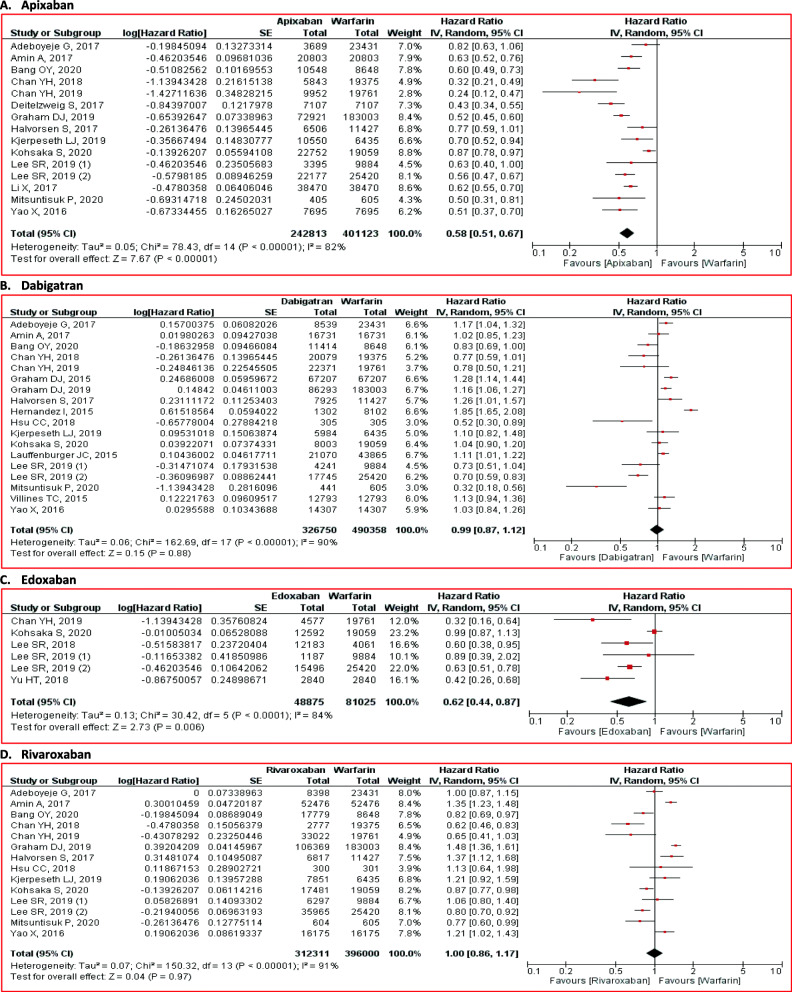
Comparison of gastrointestinal bleeding between NOACs and warfarin stratified by each agent. **A** apixaban, **B** dabigatran, **C** edoxaban, and **D** rivaroxaban. CI = confidence interval, NOACs = non-vitamin K antagonist oral anticoagulants

The analysis results for gastrointestinal bleeding were different from major bleeding and intracranial bleeding. Overall, NOACs did not significantly reduce the gastrointestinal bleeding risk (HR 0.78; 95% CI 0.58 to 1.06; p = 0.12) (Figure 2). The subgroup analysis demonstrated conflicting results. Compared with warfarin, apixaban (HR 0.58; 95% CI 0.51 to 0.67; *p* < 0.01) and edoxaban (HR 0.62; 95% CI 0.44 to 0.87; *p* < 0.01) were related with the gastrointestinal bleeding risk reduction (Fig. 7). However, the administration of dabigatran (HR 0.99; 95% CI 0.87 to 1.12; *p* =0.88) and rivaroxaban (HR 1.00; 95% CI 0.86 to 1.17; *p* = 0.97) failed to reduce gastrointestinal bleeding risk (Fig. 7). All outcomes are summarized in Table 2.

## Discussion

Our systematic review and meta-analysis study, including more than 2.2 million NVAF patients, assessed the safety and efficacy profile of warfarin and NOACs for stroke prevention in the real-world population. We analyzed the results of the real-world studies regarding anticoagulant treatment for NVAF in several countries across America, Asia, and Europe. Our study sample is smaller than the study conducted by Wang et al., which included more than 2.3 million patients [58]. However, Wang et al. only assessed the bleeding risk generally. They did not analyze the specific outcome for safety and efficacy profiles [58]. In this study, we tried to analyze the efficacy (stroke risk and all-cause mortality risk) and safety (intracranial bleeding risk, gastrointestinal bleeding risk, and major bleeding risk) profiles specifically.

The efficacy endpoint of our study included stroke risk (primary outcome) and all-cause mortality risk. In our study, NOACs effectively reduced stroke risk compared to warfarin. Our finding was similar to previous meta-analysis studies [59, 60]. In subgroup analysis, apixaban, dabigatran, and rivaroxaban also showed significant stroke risk reduction. These results supported the findings of the prior meta-analysis study [61]. However, our study provided new real-world evidence about the benefit of edoxaban for stroke risk reduction compared to warfarin. Our study also revealed that NOACs effectively reduced all-cause mortality compared to warfarin. This result was not different from the previous meta-analysis studies of RCTs [60, 62]. Our analysis on apixaban, dabigatran, and edoxaban showed the benefit of all-cause mortality risk reduction. Our results were similar to the results of previous studies [61, 63]. However, we failed to provide evidence of the advantage of rivaroxaban to reduce all-cause mortality risk.

Our study revealed that NOACs were correlated with a lower risk of intracranial bleeding and major bleeding than warfarin. Our findings supported the previous evidence from the meta-analysis of RCTs comparing NOACs and warfarin [62]. In subgroup analysis, apixaban, dabigatran, edoxaban, and rivaroxaban also showed similar results for major bleeding and intracranial bleeding. Our findings on the meta-analysis of apixaban, dabigatran, edoxaban, and rivaroxaban were consistent with the prior meta-analysis studies [61, 63, 64]. In our study, the gastrointestinal bleeding risk between NOACs and warfarin was not significantly different. Our result was different from the previous meta-analysis studies. A meta-analysis of RCTs from Ruff et al. demonstrated that NOACs were related to greater gastrointestinal bleeding risk [62]. However, in the meta-analysis of real-world studies from Chan et al., NOACs significantly decreased gastrointestinal bleeding risk [63]. Our study revealed that apixaban and edoxaban effectively reduced gastrointestinal bleeding risk. However, our study also revealed that the bleeding risks between dabigatran and rivaroxaban were not different significantly. Our results on apixaban and edoxaban supported the results of previous real-world meta-analysis studies [61, 63]. The previous meta-analysis studies on dabigatran and rivaroxaban showed conflicting results. A meta-analysis study from Chan et al. [63] showed that dabigatran and rivaroxaban did not significantly reduce the gastrointestinal bleeding risk, while a meta-analysis study from Xue et al. showed that dabigatran and rivaroxaban reduced gastrointestinal bleeding risk [61]. Those two previous meta-analyses included only the real-world data from Asian countries [61, 63]. However, our study provided real-world evidence beyond the Asian population.

Our study demonstrated that NOACS, including apixaban, dabigatran, edoxaban, and rivaroxaban, consistently revealed a significant decrease in the risk of stroke, all-cause mortality, major bleeding, and intracranial bleeding in the real-world setting. The situation in the real-world setting was quite different than in the RCTs. In RCTs, the mean time in the therapeutic range (TTR) of INR 2.0 to 3.0 ranged from 55 to 64% [10–12]. However, in most of the real-world studies, the TTR could not be recorded [24–50, 52–55, 57]. Real-world studies usually have a role in providing complementary sources of knowledge, and their results are fruitful to validate the findings from RCTs. Our study also revealed that NOACs failed to minimize the risk of gastrointestinal bleeding. The possible explanations were the unavailability of the data about: (1) patients' age; (2) the underlying gastrointestinal disease; and (3) the administration of gastroprotective agents. Moreover, the mean HAS-BLED score among the included studies also varied. That could be the essential confounding factor.

In daily clinical practice, NOACs offer more benefit than warfarin due to: (1) rapid onset of action; (2) fixed dosing; (3) few drug to drug interactions; (4) few drug to food interactions; (5) no routine laboratory monitoring; and (6) short blood-thinning effect. However, NOACs also have several drawbacks, such as the high cost and the unavailability of reversal agents [65, 66]. According to our results, we recommend NOACs as the first choice for stroke prevention in NVAF patients.

There were several limitations of our systematic review and meta-analysis study. First, almost all involved studies did not provide data about the treatment regimen’s compliance or persistence. Second, the TTR of warfarin users was not reported in almost all studies. The favorable safety and efficacy profile of NOACs might have been at least partly because of low TTR in warfarin users. Third, we did not conduct subgroup analysis comparing warfarin with a low or high dose of NOACs because of the limited available data. Fourth, among the involved studies, the precise inclusion or exclusion criteria and outcomes definitions varied. Last, even though we involved studies that reported the adjusted HR and 95% CI using either propensity score matching, propensity score weighting, or multi-variate Cox regression, the residual confounding factors with unmeasured variables could not be excluded from this study due to the characteristic of real-world data.

## Conclusions

In conclusion, our study demonstrated that NOACs had more efficacy than warfarin in preventing stroke in NVAF patients. NOACs were also related to a lower risk of all-cause mortality, intracranial bleeding, and major bleeding than warfarin. Among NOACs, apixaban and edoxaban might have a better safety and efficacy profile compared to warfarin. A head-to-head RCT that directly compares the specific type of NOACs is needed.

## Supplementary Information

**Additional file 1:.** Supplementary Table 1. Newcastle-Ottawa Scale

## Data Availability

Data used in our study were presented in the main text and supplementary material.

## Abbreviations

AF: Atrial fibrillation
CHA2DS2-VASc: Congestive heart failure, Hypertension, Age, Diabetes, previous Stroke/transient ischemic attack, VAScular disease
CI: Confidence interval
CMA: Comprehensive Meta-Analysis
CVA: Cerebrovascular accident
DOAC: Direct oral anticoagulant
GI: Gastrointestinal
HAS-BLED: Hypertension, Abnormal renal/liver function, Stroke, Bleeding history or predisposition, Labile international normalized ratio, Elderly, Drugs/alcohol concomitantly
HR: Hazard ratio
INR: International normalized ratio
NOAC: Non-vitamin K antagonist oral anticoagulant
NOS: Newcastle-Ottawa scale
NVAF: Non-valvular atrial fibrillation
PRISMA: Preferred reporting items for systematic review and meta-analysis
RCT: Randomized controlled trials
SE: Standard error
VKA: Vitamin K antagonist
VTE: Venous thromboembolism
