# Supplementary material for: Direct comparison of non-vitamin K antagonist oral anticoagulant versus warfarin for stroke prevention in non-valvular atrial fibrillation: a systematic review and meta-analysis of real-world evidences
Source: Egypt Heart J. 2021 Aug 11;73:70. doi: 10.1186/s43044-021-00194-1 (PMC8357866; doi:10.1186/s43044-021-00194-1)
Supplement: Supplementary file 1 — Additional file 1:. Supplementary Table 1. Newcastle-Ottawa Scale [file 43044_2021_194_MOESM1_ESM.docx]

Supplementary table 1. Newcastle-Ottawa Scale

| Study | Selection | | | | Comparability | Outcome | | | NOS |
| --- | --- | --- | --- | --- | --- | --- | --- | --- | --- |
|  | Representativeness of the exposed cohort | Representativeness of the exposed cohort | Ascertainment of exposure | Demonstration that outcome of interest was not present at start of study | Comparability of cohorts on the basis of the design or analysis controlled for confounders | Assessment of outcome | Was follow-up long enough for outcomes to occur | Adequacy of follow-up of cohorts |  |
| Adeboyeje G, 2017 | 1 | 1 | 1 | 0 | 1 | 1 | 1 | 1 | 7 |
| Amin A, 2017 | 1 | 1 | 1 | 0 | 1 | 1 | 1 | 1 | 7 |
| Bang OY, 2020 | 1 | 1 | 1 | 0 | 2 | 1 | 1 | 1 | 8 |
| Cha MJ, 2017 | 1 | 1 | 1 | 1 | 1 | 1 | 1 | 1 | 8 |
| Chan YH, 2018 | 1 | 1 | 1 | 0 | 1 | 1 | 1 | 1 | 7 |
| Chan YH, 2019 | 1 | 1 | 1 | 0 | 2 | 1 | 1 | 1 | 8 |
| Cho MS, 2019 | 1 | 1 | 1 | 0 | 2 | 1 | 1 | 1 | 8 |
| Coleman CI, 2017 | 1 | 1 | 1 | 0 | 2 | 1 | 1 | 1 | 8 |
| Costa OS, 2020 | 1 | 1 | 1 | 0 | 2 | 1 | 1 | 1 | 8 |
| Deitelzweig S, 2017 | 1 | 1 | 1 | 0 | 1 | 1 | 1 | 1 | 7 |
| Graham DJ, 2015 | 1 | 1 | 1 | 0 | 2 | 1 | 1 | 1 | 8 |
| Graham DJ, 2019 | 1 | 1 | 1 | 0 | 2 | 1 | 1 | 1 | 8 |
| Halvorsen S, 2017 | 1 | 1 | 1 | 0 | 1 | 1 | 1 | 1 | 7 |
| Hernandez I, 2015 | 1 | 1 | 1 | 0 | 2 | 1 | 1 | 1 | 8 |
| Hsu CC, 2018 | 1 | 1 | 1 | 0 | 1 | 1 | 1 | 1 | 7 |
| Huybrechts KF, 2020 | 1 | 1 | 1 | 0 | 2 | 1 | 1 | 1 | 8 |
| Kjerpeseth LJ, 2019 | 1 | 1 | 1 | 0 | 1 | 1 | 1 | 1 | 7 |
| Kohsaka S, 2020 | 1 | 1 | 1 | 0 | 2 | 1 | 1 | 1 | 8 |
| Larsen TB, 2016 | 1 | 1 | 1 | 0 | 2 | 1 | 1 | 1 | 8 |
| Lauffenburger JC, 2015 | 1 | 1 | 1 | 0 | 2 | 1 | 1 | 1 | 8 |
| Lee SR, 2018 | 1 | 1 | 1 | 1 | 2 | 1 | 1 | 1 | 9 |
| Lee SR, 2019 (1) | 1 | 1 | 1 | 1 | 2 | 1 | 1 | 1 | 9 |
| Lee SR, 2019 (2) | 1 | 1 | 1 | 1 | 2 | 1 | 1 | 1 | 9 |
| Li X, 2017 | 1 | 1 | 1 | 0 | 2 | 1 | 1 | 1 | 8 |
| Lip YH, 2016 (1) | 1 | 1 | 1 | 0 | 1 | 1 | 1 | 1 | 7 |
| Lip YH, 2016 (2) | 1 | 1 | 1 | 0 | 1 | 1 | 1 | 1 | 7 |
| Maura G, 2015 | 1 | 1 | 1 | 1 | 2 | 1 | 1 | 1 | 9 |
| Mitsuntisuk P, 2020 | 1 | 1 | 1 | 0 | 2 | 1 | 1 | 1 | 8 |
| Nielsen PB, 2017 | 1 | 1 | 1 | 0 | 2 | 1 | 1 | 1 | 8 |
| Rutherford OCW, 2020 | 1 | 1 | 1 | 0 | 2 | 1 | 1 | 1 | 8 |
| Staerk L, 2017 | 1 | 1 | 1 | 0 | 1 | 1 | 1 | 1 | 7 |
| Villines TC, 2015 | 1 | 1 | 1 | 0 | 1 | 1 | 1 | 1 | 7 |
| Yao X, 2016 | 1 | 1 | 1 | 0 | 1 | 1 | 1 | 1 | 7 |
| Yu HT, 2018 | 1 | 1 | 1 | 0 | 2 | 1 | 1 | 1 | 8 |

NOS = Newcastle-Ottawa Scale
